# Role of Parathyroid Hormone-Related Protein Signaling in Chronic Pancreatitis

**DOI:** 10.3390/cancers7020826

**Published:** 2015-06-18

**Authors:** Miriam Falzon, Vandanajay Bhatia

**Affiliations:** Department of Pharmacology and Toxicology, University of Texas Medical Branch, Galveston, TX 77555, USA; E-Mail: vabhatia@utmb.edu

**Keywords:** parathyroid hormone-related protein, alcohol, acinar cells, stellate cells, cytokines, extracellular matrix

## Abstract

Chronic pancreatitis (CP), a progressive inflammatory disease where acini are destroyed and replaced by fibrous tissue, increases the risk for pancreatic cancer. Risk factors include alcohol, smoking, and obesity. The effects of these risk factors are exacerbated in patients with mutations in genes that predispose to CP. The different environmental and genetic factors produce the same clinical phenotype; once CP develops, disease course is the same regardless of etiology. Critical questions still need to be answered to understand what modifies predisposition to develop CP in persons exposed to risk factors. We postulate that risk factors modulate endogenous pathways, with parathyroid hormone-related protein (PTHrP) signaling being one such pathway. In support, PTHrP levels are elevated in mice treated with alcohol, and in mouse models of cerulein- and pancreatic duct ligation-induced CP. Disrupting the *Pthrp* gene in acinar cells exerts protective effects (decreased edema, histological damage, amylase and cytokine release, and fibrosis) in these CP models. PTHrP levels are elevated in human CP. Currently, CP care lacks specific pharmacological interventions. Targeting PTHrP signaling may present a novel therapeutic strategy that inhibits pancreatic inflammation and fibrosis, especially since the risk of developing pancreatic cancer is strongly associated with duration of chronic inflammation.

## 1. Inflammation and Pancreatic Cancer

Pancreatic cancer is the fourth leading cause of cancer-related deaths worldwide, and the five-year relative survival rate is ~7% [1]. This is not surprising as this cancer is difficult to predict, detect, and diagnose, and is resistant to all current treatments except early surgery [2]. Distant metastasis is frequently present at time of diagnosis. Clinically, >80% of patients present with an unresectable tumor with distant organ metastasis, and the five-year survival rate is <6% [3]. This highlights the importance of understanding the etiology of pancreatic cancer. While a number of familial syndromes are associated with this cancer, only ~10% of patients present with a strong family history [4]. Chronic/hereditary pancreatitis is a recognized risk factor for pancreatic cancer, and chronic pancreatitis (CP) and pancreatic cancer share many of the same risk factors, including alcohol abuse, smoking, and obesity [5,6,7].

Multiple lines of evidence from genetic, pharmacological, and epidemiological studies have established a connection between inflammation and cancer, with many cancers arising from sites of chronic irritation, infection, or inflammation [8]. The molecular mechanisms via which inflammation promotes cancer development are still being uncovered, and may be different in different tissues [8]. Recent research has clearly demonstrated a significant role for inflammation in pancreatic cancer, and the link between inflammation and pancreatic ductal adenocarcinoma (PDA) are well established [9].

## 2. Etiology and Pathology of Chronic Pancreatitis

One of the leading causes of pancreatic inflammation is CP, a progressive, destructive inflammatory process that is characterized by chronic inflammation, tissue destruction, and fibrosis [10]. Irrespective of the etiology, pancreatitis involves a common cascade of events. Initial acinar cell injury causes aberrant zymogen secretion and premature activation, leading to tissue auto-digestion, generation of an inflammatory response, focal necrosis, and fibrosis [10,11,12]. With recurrent episodes of acute pancreatitis (AP), the pancreas does not adequately recover from the repeated injury, thereby perpetuating the conditions of chronic inflammation and irreversible fibrosis [10,11]. Approximately 20% of patients with AP develop systemic inflammation and multiple organ failure. Epidemiological, etiological, and experimental data indicate that CP results from the accumulated damage incurred during repeated bouts of AP (recurrent acute pancreatitis or RAP). Data indicate that AP progresses to RAP and then to CP in a disease continuum. The necrosis-fibrosis model postulates that recurring inflammation and necrosis as a result of multiple episodes of AP leads to atrophy and fibrosis (reviewed in [13]). The more recent sentinel acute pancreatitis event (SAPE) model divides the pathogenesis of CP into three sequential stages: pre-acute pancreatitis, the initial or sentinel attack of AP (first hit), and the progression phase (second hit) (reviewed in [14]). In the pre-acute stage, the pancreas is exposed to risk factors for CP (such as heavy alcohol use, smoking, obesity, and genetic risk). In the sentinel event, the first episode of AP occurs as a result of activation of the immune system. Chronic inflammation and pancreatic stellate cell (PSC) activation occur in this phase. The third or progression stage is dependent on factors that drive the immune response through a variety of possible stressors, including mutations in the *PRSS1* gene (the cause of hereditary pancreatitis), metabolic stress due to excessive alcohol consumption and/or smoking, and continued exposure to factors such as alcohol that cause recruitment of inflammatory cells, which in turn increase levels of anti-inflammatory cytokines and drive fibrosis. The presence of these stressors may push the pancreas towards continued inflammation and fibrosis, and facilitates RAP by lowering the threshold for initiating trypsinogen activation in acinar cells, impairing pancreatic duct cell secretion, or modulating the immune or fibrosis response [14]. In the absence of stressors, the pancreas can return to normal. While the natural history of CP may differ depending on the various environmental and genetic factors, disease outcome does not. Once CP develops, disease course is the same regardless of etiology [15].

## 3. Increased Risk for Pancreatic Cancer in Patients with Chronic Pancreatitis

CP has a strong negative effect on the quality of life. Patients with CP experience chronic abdominal pain, impaired digestion, malnutrition, anorexia, diabetes, and disease-related complications such as pseudocyst formation [16]. There is no cure for CP, and treatment options are limited to supportive care and symptom palliation, including management of chronic pain, replacement of digestive enzymes, vitamin supplementation, and glucose control [17]. As the disease progresses, patients may require more invasive interventions, ranging from endoscopic stenting of strictures to surgical bypass procedures or even total pancreatectomy [18].

In addition to these adverse effects on quality of life, patients with CP have a significant added risk of developing pancreatic cancer. In fact, the one consistent risk factor for pancreatic cancer is CP [19]. An international cooperative study initiated in 1993 [20] established a cumulative risk of PDA in subjects with CP of 1.8% after 10 years and 4.0% after 20 years, with a standardized incidence ratio of 14.4. In a later international epidemiology study [21], eight cases of proven pancreatic cancer were observed against a background expected number of 0.15, giving a relative risk of 53 times normal. Multiple epidemiologic studies have since confirmed these observations [2,22]. The relationship between CP and PDA is further strengthened in studies of patients who develop tropical pancreatitis (a major cause of childhood CP in tropical regions, associated with mutations in the *SPINK1* gene), or childhood-onset CP, which is associated with genetic factors such as mutations in *PRSS1* and *CFTR* genes [23,24]. These mutations all result in defects in the mechanisms that protect the pancreas from premature trypsinogen activation [24].

Presently CP disease is not reversible, and there are no targeted therapies for this disease. Given the severity of CP disease and its strong potential to lead to PDA, it is imperative to identify endogenous signaling pathways that modify the response of the pancreas after exposure to risk factors associated with the disease.

## 4. Risk Factors of CP

Alcohol continues to be the single most common cause of CP, with an attributable risk of ~40%. Individuals with a history of alcoholism have an ~4-fold greater risk of developing pancreatitis (reviewed in [25]). Pancreatic damage in chronic alcoholics is evident before the onset of clinical pancreatitis; at autopsy, changes consistent with CP are present in 5%–10% of heavy alcohol users, even in the absence of clinical symptoms of pancreatitis [26,27]. Alcohol appears to increase the sensitivity of the pancreas to injury from other factors, whether they are genetic or environmental (reviewed in [25,28,29]). Cigarette smoking is an independent risk factor for pancreatitis, and smoking and alcohol may exert synergistic damaging effects on the pancreas [30,31,32]. Diet, particularly that which is rich in protein and fat, may also contribute to the modulation and progression of alcoholic pancreatitis [33,34].

While alcohol is a significant risk factor for pancreatitis, only 10%–15% of clinically documented alcoholics develop symptomatic disease (reviewed in [26]). The lack of a homogeneous, dose-dependent effect of alcohol on the exocrine pancreas in humans is reflected in animal models of alcohol-induced ethanol pancreatitis. Ethanol feeding alone does not produce CP, and inflammation and fibrosis are only observed when ethanol feeding is combined with other injurious agents, such as lipopolysaccharide or a low dose of the cholecystokinin (CCK) analog cerulein [35]. The most informative use of the alcohol model has been its use in studies of sensitization to other injurious agents.

The foregoing discussion illustrates that critical questions still need to be answered to understand what modifies a person’s predisposition to develop CP in response to exposure to risk factors. It is still not fully understood why disease severity varies widely between individuals and why the disease only progresses in some people, despite the presence of the same risk factors. Exposure to risk factors may modulate endogenous signaling protective pathways. The degree to which these endogenous pathways are modulated after exposure to risk factors may determine the extent of pancreatic damage and whether there is disease progression or resolution in a given individual. The parathyroid hormone-related protein (PTHrP) signaling pathway may be one such pathway.

## 5. Parathyroid Hormone-Related Protein Biology

Parathyroid hormone-related protein (PTHrP) exerts multiple effects in normal physiology and disease states. The protein is only detected in the circulation of normal subjects during pregnancy and lactation, and in cancer patients with the accompanying syndrome of humoral hypercalcemia of malignancy (HHM). In normal subjects and in cancer patients in the absence of HHM, PTHrP exerts its effects via autocrine/paracrine and intracrine pathways. PTHrP is expressed by most fetal and adult tissues, and the mature PTHrP species is post-translationally processed to N-terminal, mid-region, and C-terminal secretory forms (reviewed in [36,37]). PTHrP shares close homology in the N-terminal sequence to parathyroid hormone (PTH), and N-terminal fragments of both PTH and PTHrP interact with the same receptor, the type 1 PTH/PTHrP receptor (PTH1R), a G protein-coupled receptor (GPCR). N-terminal PTHrP exerts its autocrine/paracrine effects by interacting with this receptor [36,38], and has been shown to play a role in the skeleton (delays chondrocyte differentiation), teeth (promotes the formation of osteoclasts), mammary gland (required for breast development), placenta (promotes calcium transport from mother to fetus and is required to maintain normal fetal calcium concentrations), smooth muscle (relaxes stretched muscle), and cardiovascular system (acts as vasodilator in resistance vessels) [36,37]. Mid-region peptides stimulate placental calcium transport and modulate renal bicarbonate handling, while C-terminal fragments are thought to inhibit osteoclast function and stimulate osteoblast proliferation (reviewed in [37]). Receptors for mid-region and C-terminal peptides have not been identified to date. Intracrine PTHrP action is mediated via a bipartite nuclear localization signal (NLS) and involves its translocation to the nucleus or nucleolus [39,40,41]. PTHrP increases cell proliferation, migration, and invasion, and decreases apoptosis [42,43,44]. PTHrP is normally expressed in pancreatic islets, where it increases cell proliferation and insulin release, and inhibits apoptosis, through a PKC-mediated pathway [45,46,47]. PTHrP expression is very low in acinar cells and PSCs within the exocrine pancreas, and no function for PTHrP has been reported in the exocrine pancreas under basal conditions.

## 6. Increased PTHrP Levels after Acinar Cell and PSC Injury Lead to Elevated Cytokine and ECM Protein Levels

The inflammatory response is initiated by injured acinar cells that produce inflammatory mediators, including cytokines and adhesion molecules, ultimately leading to systemic complications [48]. Alcohol regulates cytokine levels in a number of tissues [49] and induces acinar cell injury and PSC activation at the cellular level [50,51,52]. We postulate a role for PTHrP in alcohol-induced pancreatic damage, in that exposure of acinar cells to alcohol resulted in a robust increase in PTHrP mRNA and protein levels [53]. The CCK analog cerulein also induces acinar cell damage [48]. Treatment of acinar cells with cerulein also increased PTHrP expression [53]. In turn, exogenous treatment of acinar cells with the active PTHrP moiety, PTHrP (1–36) increased levels of the proinflammatory mediators interleukin (IL)-6 and intercellular adhesion molecule-1 (ICAM-1) in acinar cells [53]. PTHrP also exerts pro-inflammatory effects in the injured kidney, and in atherosclerosis and rheumatoid arthritis [54,55,56].

Alcohol and cerulein also induce PTHrP expression in PSCs [53]; these cells are the major mediators of the fibrosis response in CP injury and inflammation [57]. PSC activation is accompanied by an increase in extracellular matrix (ECM) proteins, the leading cause of progressive fibrosis and ultimately pancreatic insufficiency [57]. PTHrP (1–36) increased levels of collagen I and fibronectin in these cells [53]. Both acinar cells and PSCs express PTH1R [53,58], indicating the feasibility of an autocrine and/or paracrine role for PTHrP in these cells. These data collectively demonstrate enhanced PTHrP expression in response to acinar and PSC damage, and that PTHrP exerts direct effects in both acinar cells and PSCs.

Tumor necrosis factor-α (TNF-α) is produced during the initial inflammatory response in many disease states, and initiates multiple downstream events, including release of other cytokines, chemokines, and endothelial adhesion molecules [59]. TNF-α levels are elevated in the serum of patients with pancreatitis and in animal models of pancreatitis [60,61,62,63]. Pancreatic acinar cells produce, release, and respond to TNF-α [64]. Exposure of acinar cells to alcohol results in increased TNF-α levels, and alcohol exacerbates acinar cell-mediated inflammatory responses [63]. TNF-α also plays a role in cerulein-induced pancreatitis [64]. TNF-α in turn upregulates multiple cytokines and chemokines, including IL-8, ICAM-1, monocyte chemoattractant protein-1 (MCP-1), and matrix metalloproteinase-1 (MMP-1) [65]. Treating acinar cells with TNF-α significantly increased PTHrP mRNA levels [66]. Thus, PTHrP may also be a cytokine upregulated in response to the acinar cell injury-induced increase in TNF-α. In fact, the hypothesis that PTHrP is a cellular cytokine was introduced by Martin *et al.*, in 1997 [67]. In addition, PTHrP levels may also be increased directly in response to acinar cell injury independent of TNF-α.

Transforming growth factor-β (TGF-β) is also secreted by acinar cells and PSCs in response to injury and plays a key role in both the inflammatory and fibrotic responses observed in pancreatitis [68,69,70,71]. Inhibition of TGF-β action protects the pancreas against chronic injury by preventing acinar cell apoptosis [72]. The molecule plays a key role in PSC activation and increases ECM deposition, leading to fibrosis [73,74,75]. In humans, TGF-β levels are elevated in both AP and CP [76,77,78]. A positive feedback loop linking TGF-β and PTHrP was first described in breast cancer, where PTHrP was identified as an effector of TGF-β in bone metastases [79]. Later studies have shown that TGF-β regulates PTHrP expression in multiple cell types, including chondrocytes, and hepatocellular carcinoma and hepatoma cells [80,81]. TGF-β upregulated PTHrP levels in both acinar cells and PSCs [66]. TGF-β may thus function upstream of PTHrP to regulate pro-inflammatory and pro-fibrotic responses.

PTHrP upregulated directly or indirectly as a result of pancreatic damage may function as an intermediate to promote the effects of alcohol on pro-inflammatory cytokine and chemokine levels. This hypothesis was investigated using the PTHrP moiety PTHrP (7–34), which functions as a competitive inhibitor of PTHrP signaling via the PTH1R. In support, the alcohol- and cerulein-induced upregulation of IL-6 and ICAM-1 levels was suppressed by pre-treatment with the PTH1R antagonist PTHrP (7–34). PTHrP (7–34) also blocked the stimulatory effects of alcohol and cerulein on procollagen I levels in PSCs [53,58]. PTHrP may therefore participate in the early stages of pancreatic injury to initiate a cascade of events that ultimately leads to pro-inflammatory cytokine release, as well as in the later events that are associated with development of fibrosis. Activation of the NF-κB signaling pathway may play a significant role in PTHrP’s effects, in that exogenous PTHrP (1–36) increased NF-κB activity in acinar cells [58]. NF-κB in turn regulates the transcription of several genes that are involved in the inflammatory response of pancreatitis [82,83,84,85,86,87]. TNF-α regulates NF-κB activity [88], and multiple pro-inflammatory cytokines, including IL-6, are regulated via this pathway [88,89].

The effects of PTHrP on mouse acinar cells and PSCs were reproduced in human cells, which were isolated from discarded human pancreatic tissue obtained from surgical resection from cadaveric organ donors [58]. Treatment with PTHrP (1–36) increased IL-6 and ICAM-1 levels in acinar cells and procollagen I mRNA levels in PSCs [58]. Blocking PTHrP action with PTHrP (7–34) suppressed the stimulatory effects of cerulein and alcohol on IL-6 and ICAM-1 levels in acinar cells and procollagen I levels in PSCs ([58,66]). It should be pointed out that there has been some controversy regarding the presence of functional CCK1 receptors, which mediate the direct effects of cerulein, in human acinar cells. While some studies have reported that these cells might lack functional CCK1 receptors [90,91], a more recent study has shown that physiological concentrations of CCK directly stimulate amylase release by isolated human pancreatic acinar cells [92]. Cerulein also signals by an indirect pathway, involving interaction with the CCK1 receptor expressed in afferent neurons that regulate pancreatic secretion via a vagal–vagal loop, with the final mediator being acetylcholine [93,94]. The human acinar cells used by Bhatia *et al.* [58] were isolated as cell clusters, and therefore it is possible that the observed effects of cerulein in these cells may be indirect and therefore mediated by the afferent neurons present in the acini. Irrespective of whether cerulein regulates PTHrP expression directly or indirectly, PTHrP still plays a role in the human acinar cells. Human PSCs express the CCK1 receptor and respond to CCK [95]. These results validate the value of continuing research into the role of PTHrP in CP.

## 7. PTHrP May Play a Role in Sensitizing Pancreatic Cells to the Effects of Alcohol

Alcohol induces a dose-dependent sensitization of the pancreas in response to CCK- or cerulein-mediated hyperstimulation of acinar cells, leading to cell damage both *in vitro* and *in vivo* [51,96,97]. Studies utilizing isolated cells indicate that PTHrP may play a role in the sensitizing effects of alcohol, where the elevated PTHrP levels observed after alcohol treatment may be sensitizing the pancreas such that its ability to adapt and respond to stressful stimuli is diminished. This may lead to RAP under conditions of further insult. PTHrP may therefore function as an early response gene that mediates the critical acute phase response in AP, thereby participating in the sentinel phase of the SAPE hypothesis model. In support, co-treating acinar cells and PSCs with alcohol plus cerulein upregulated PTHrP expression at doses where the individual compounds exerted minimal or no effect [53]. Elevated PTHrP expression was accompanied by increased expression of IL-6 and procollagen I, again at doses where alcohol or cerulein alone had no effect [53]. PTHrP upregulation at the pre-AP stage may therefore initiate a cascade of events that ultimately leads to the inflammatory and fibrotic response associated with RAP, eventually leading to CP.

## 8. PTHrP Levels Are Increased in Mouse Models of AP and CP 

PTHrP levels are also elevated in *in vivo* models of pancreatic damage. Treatment with supraphysiologic doses of cerulein is a widely-employed animal model of AP [35]. This model of pancreatic damage simulates that seen in human edematous pancreatitis, and is evident as dysregulation of digestive enzyme production and cytoplasmic vacuolization, acinar cell death, edema formation, and infiltration of inflammatory cells into the pancreas [48]. Using this model, Bhatia *et al.* [53] showed a robust increase in PTHrP expression in both acinar cells and PSCs. Short-term exposure to ethanol *in vivo* also increased PTHrP levels in acinar cells and PSCs [53], indicating a possible role for PTHrP in the inflammatory and fibrotic response which accompanies alcohol-induced pancreatitis.

Multiple animal models of CP have been developed, including repetitive acute pancreatitis, duct ligation models, alcohol feeding, and genetic models [98]. The repetitive cerulein administration model is one of the most widely used models of CP. In this model, pancreatic damage is followed by periods of recovery, thereby simulating the process of RAP in humans [98,99]. Since blocking pancreatic secretion can also induce acinar cell necrosis, the mouse model of pancreatic duct ligation (PDL) is relevant for obstruction-induced forms of CP. Pancreatic duct obstruction due to stenosis and/or intraductal stones is an important etiologic factor in development of CP in humans [98,99]. In both models, pancreatic damage leads to fibrotic remodeling of the pancreatic parenchyma and eventually to pancreatic insufficiency [100,101,102]. Pancreatic PTHrP levels were elevated in both the cerulein- and PDL-induced models of pancreatic damage [58]. Moreover, immunohistochemical analysis revealed higher PTHrP levels in pancreata from patients with CP compared to those from normal subjects [58]. These findings reinforce those obtained using isolated cells and establish PTHrP as a novel mediator of the inflammation and fibrosis associated with CP.

## 9. PTHrP May Function as a CP Modifier Molecule

**Figure 1:**
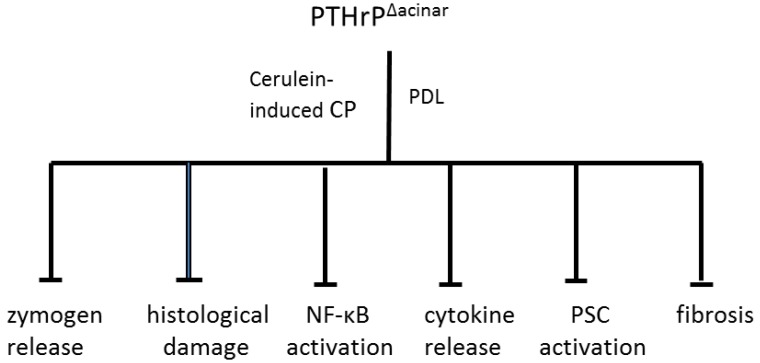
Protective effect of *Pthrp* gene deletion in acinar cells (PTHrP^Δacinar^) in mouse models of cerulein-induced and pancreatic duct ligation-induced (PDL) pancreatitis. Acinar cell-specific targeted disruption of the *Pthrp* gene was achieved using Cre-LoxP technology and the acinar cell-specific elastase promoter. *Cre* recombinase was activated by injection with tamoxifen. For the cerulein-induced CP model, mice received five injections of cerulein (50 µg·kg^−1^) at 1 h intervals three days a week for three weeks, and were sacrificed four days after the last injection. For the PDL model, the splenic duct was ligated and mice were sacrificed two days later. PTHrP^Δacinar^ significantly inhibited pancreatic damage, including zymogen release, histological damage, NF-κB activation, cytokine release, PSC activation (assessed by monitoring α-SMA levels) and fibrosis (assessed using collagen as marker).

As noted earlier, susceptibility for developing CP may be dependent on modulation of endogenous signaling pathways activated as a result of pancreatic cell injury. The role of acinar cell-secreted PTHrP in modulating pancreatic injury was therefore investigated using mice with acinar cell-specific targeted disruption of the *Pthrp* gene (PTHrP^∆acinar^). These mice were generated using Cre-LoxP technology [103,104,105] and the acinar cell-specific elastase promoter [106]. PSCs from these mice still expressed PTHrP [58]. PTHrP^∆acinar^ exerted protective effects in both the chronic repetitive cerulein and PDL models of pancreatitis, evident as decreased edema, histological damage, and serum amylase levels (Figure 1; [58]). PTHrP^∆acinar^ also inhibited PSC activation, assessed by monitoring α-smooth muscle actin (α-SMA) levels and collagen deposition (Figure 1; [58]). The effects of chronic repetitive cerulein and PDL on pancreatic IL-6 levels were also significantly attenuated in PTHrP^∆acinar^ mice, supporting the hypothesis that PTHrP may function by upregulating pro-inflammatory cytokine release. Moreover, effects of cerulein on IL-6 expression and NF-κB activity were attenuated in acinar cells isolated from mice with *Pthrp* gene knockout, as were the cerulein- and carbachol-induced elevations in amylase secretion [58]. In combination with previous findings showing a suppressive effect of PTHrP (7–34) on the upregulatory effects of cerulein on IL-6 levels *in vitro*, these data strongly support a pivotal role for PTHrP directly at the acinar cell level. These data are significant as acinar cells are the initial sites of injury in CP. Chronic acinar cell injury is tightly associated with inflammation and subsequent fibrosis by activating PSCs [57].

## 10. Conclusions

Pancreatic cancer has a high mortality rate and is one of the top causes of cancer deaths [1]. The one consistent risk factor for pancreatic cancer is CP [19,22]. CP is thought to result from the accumulated damage incurred during RAP [13]. Currently, there are no targeted therapies for CP and prevention or reversal of CP disease is not achievable. Targeting endogenous signaling pathways which may enhance the sensitivity of the pancreas exposed to risk factors may prevent development of RAP, and therefore CP. PTHrP may be one such endogenous signaling pathway that is activated as a result of pancreatic cell injury, and may therefore be a potential target for therapeutic strategies designed to reduce pancreatic inflammation and ultimately fibrosis.

**Figure 2:**
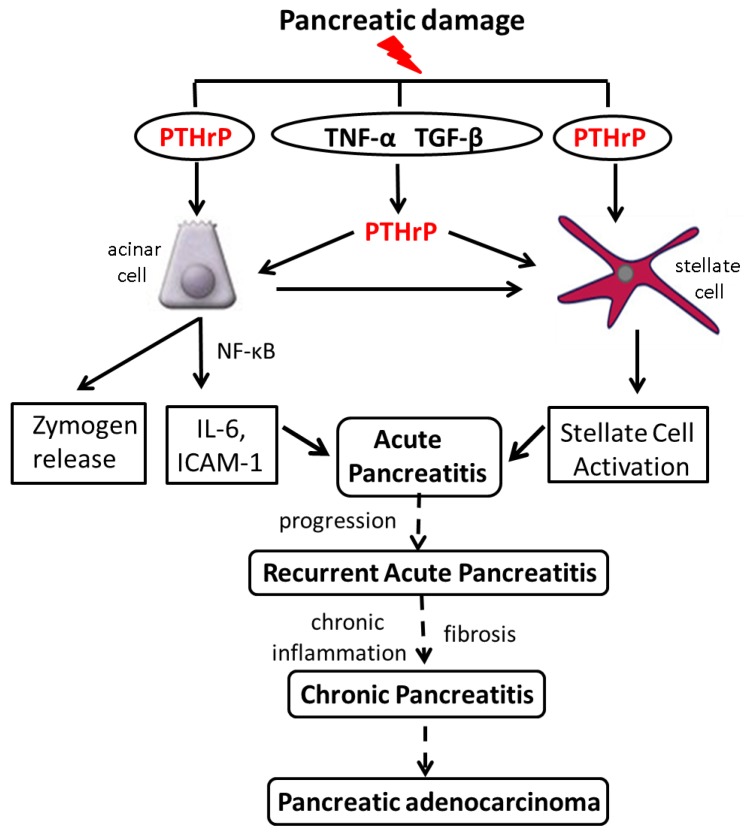
Working model of the pathways by which PTHrP functions in the exocrine pancreas. Pancreatic damage induced by injurious agents such as alcohol results in upregulation of PTHrP expression. PTHrP may be targeted directly at the transcriptional and/or post-transcriptional levels, or may be upregulated downstream of cytokines whose levels are elevated as a result of pancreatic damage, including TNF-α and TGF-β. PTHrP released by acinar cells in turn functions via an autocrine and/or paracrine pathway to induce cytokine and chemokine release, leading to an inflammatory response. Acinar cell-secreted PTHrP may also function by a paracrine pathway to activate PSCs. PSCs also release PTHrP in response to injury, leading to their activation. The net result is development of Acute Pancreatitis. Further exposure to risk factors, accompanied by release of PTHrP, would perpetuate the pro-inflammatory and pro-fibrotic response. These multiple episodes of acute pancreatitis (Recurrent Acute Pancreatitis) may eventually lead to Chronic Pancreatitis, with chronic inflammation and scarring. Since a history of pancreatitis is a significant risk factor for PDA, preventing or limiting development of CP through prophylactic use of inhibitors of the PTHrP signaling pathway may reduce the risk for this cancer.

Acinar cell and PSC damage induced following exposure to alcohol and cerulein increases PTHrP levels (Figure 2). These effects may be direct; both alcohol and cerulein have been reported to regulate multiple genes at the transcriptional level [107,108]. Alternatively, PTHrP may be upregulated downstream of cytokines known to be involved in the initial response of the pancreas to injury, such as TNF-α and TGF-β. PTHrP secreted by acinar cells in turn regulates cytokine, chemokine and zymogen release, and may function via a paracrine pathway to regulate PSCs. In support, PSC activation is suppressed in PTHrP^Δacinar^ mice. PSCs also secrete PTHrP in response to injury, resulting in increased ECM protein levels (Figure 2). These effects of PTHrP may ultimately result in RAP and CP.

The use of human acinar cells and PSCs has demonstrated parallel effects of PTHrP in human and rodent cells. Therefore, development of strategies aimed at targeted delivery of antagonists of PTHrP or PTH1R in acinar cells may present a novel therapeutic strategy aimed at preventing pancreatic inflammation and fibrosis, irrespective of the primary risk factor(s) involved. Suppression of PTHrP expression has been shown to be a promising approach for anti-cancer strategies in chondrosarcoma, thyroid cancer, medulloblastoma, adrenocortical cancer, oral squamous, renal, colon, prostate, and triple-negative breast cancer cells (reviewed in [109,110]). Drugs that suppress PTHrP expression, such as the farnesyltransferase inhibitor manumycin and guanine nucleotide analogs [111,112], may also exert a protective effect in the pancreas exposed to risk factors. Suppression of PTHrP expression would ultimately prevent development of CP in patients prone to RAP. Since the presence of CP is a consistent risk factor for pancreatic cancer, prevention of CP may hinder or halt development of this devastating disease.
